# The mitochondrial genome of the hexactinellid sponge *Aphrocallistes vastus*: Evidence for programmed translational frameshifting

**DOI:** 10.1186/1471-2164-9-33

**Published:** 2008-01-23

**Authors:** Rafael D Rosengarten, Erik A Sperling, Maria A Moreno, Sally P Leys, Stephen L Dellaporta

**Affiliations:** 1 Department of Molecular, Cellular and Developmental Biology, Yale University, New Haven, CT 06520-8104, USA; 2 Department of Geology and Geophysics, Yale University, New Haven, CT 06520, USA; 3 Department of Biological Sciences, University of Alberta, Edmonton, Alberta, T6G 2E9, Canada

## Abstract

**Background:**

Mitochondrial genomes (mtDNA) of numerous sponges have been sequenced as part of an ongoing effort to resolve the class-level phylogeny of the Porifera, as well as to place the various lower metazoan groups on the animal-kingdom tree. Most recently, the partial mtDNA of two glass sponges, class Hexactinellida, were reported. While previous phylogenetic estimations based on these data remain uncertain due to insufficient taxon sampling and accelerated rates of evolution, the mtDNA molecules themselves reveal interesting traits that may be unique to hexactinellids. Here we determined the first complete mitochondrial genome of a hexactinellid sponge, *Aphrocallistes vastus*, and compared it to published poriferan mtDNAs to further describe characteristics specific to hexactinellid and other sponge mitochondrial genomes.

**Results:**

The *A. vastus *mtDNA consisted of a 17,427 base pair circular molecule containing thirteen protein-coding genes, divergent large and small subunit ribosomal RNAs, and a reduced set of 18 tRNAs. The *A. vastus *mtDNA showed a typical hexactinellid nucleotide composition and shared a large synteny with the other sequenced glass sponge mtDNAs. It also contained an unidentified open reading frame and large intergenic space region. Two frameshifts, in the *cox3 *and *nad6 *genes, were not corrected by RNA editing, but rather possessed identical shift sites marked by the extremely rare tryptophan codon (UGG) followed by the common glycine codon (GGA) in the +1 frame.

**Conclusion:**

Hexactinellid mtDNAs have shown similar trends in gene content, nucleotide composition, and codon usage, and have retained a large gene syntenty. Analysis of the mtDNA of *A. vastus *has provided evidence diagnostic for +1 programmed translational frameshifting, a phenomenon disparately reported throughout the animal kingdom, but present in the hexactinellid mtDNAs that have been sequenced to date.

## Background

The Phylum Porifera (sponges) is currently divided into three extant classes – the Hexactinellida, Demospongiae, and Calcarea – and one fossil class – the Archaeocyatha [1]. Hexactinellids differ from the canonical sponge body plan in lacking discrete motile cells (see [1,2] for a detailed review). The tissue of hexactinellids forms a continuous multinucleate syncytium. Cellular components exist, but all "cells" are connected by cytoplasmic bridges to one-another and to the syncytium. Choanocytes are branched; collar-flagella units ("collar bodies") form as enucleate buds, several arising from a single nucleated choanoblast [2-4]. The distinct tissue organization was considered important enough to warrant separation of hexactinellids from other Porifera at the subphylum level – Symplasma for the Hexactinellida and Cellularia for the Demospongiae and Calcarea [5]. In no other animal is the tissue so inclusively connected in a giant syncytium, making the hexactinellid body construction unique among Porifera, as well as all Metazoa.

The Porifera has long been considered the earliest branching group of the metazoan crown, based on both morphological and molecular evidence, although the precise relationships of the lower metazoan phyla remains uncertain [6-11]. One on-going approach to resolve the overall metazoan phylogeny, as well as the problematic class-level relationships among the sponges, has been comparative analysis of mitochondrial genomes (mtDNA). To date, this effort has seen the sequencing and description of five complete demosponge mtDNAs [GenBank: NC_006894, NC_006990, NC_006991, NC_008944, NC_009090], and most recently, that of two partial hexactinellid sponge mtDNAs [GenBank: EF537576, EF537577]. Phylogenetic estimations based on concatenated mitochondrial protein sequences have been sensitive to taxon sampling, outgroup choice and algorithm implementation. These trees reveal artifacts likely due to variable rates of molecular evolution, such that placozoans, cnidarians, and demosponges are recovered as a monophyletic clade, with hexactinellids the sister group of bilaterians [12-16]. The tree topologies may become more stable, and consistent with plausible hypotheses of the evolution of morphological traits, as the mtDNA of additional taxa are added to the data set.

While sequence-based mtDNA comparisons of the lower metazoa have not been phylogenetically definitive, the efforts have dispelled some commonly held myths and revealed some general characteristics of animal mtDNA. Placozoan mtDNA, for example, is twice as large as most animal molecules, ranging from 32 to 43 kilobases, and retains various traits of the non-metazoan outgroups such as substantial intergenic space, introns and large open reading frames less commonly found in other animal mtDNA [12,17]. On the other hand, demosponge mtDNA is more typical of other animals – compact molecules, between 18 and 25 kb, with few or no introns, little intergenic space, and coding for twelve to fourteen respiratory chain proteins and two ribosomal RNAs [13,15,16,18,19]. Two partial hexactinellid mitochondrial genomes, those of *Sympagella nux *(Order Lyssacinosida, Family Caulophacidae) and *Iphiteon panicea *(Order Hexactinosida, Family Dacylocalycidae), have recently been reported [14]. These genomes were found to have similar protein-coding gene content as the published demosponges, but had several features such as tRNA structure and content more similar to that of bilaterians [14]. The current paper reports on the complete mtDNA sequence of the hexactinellid sponge *Aphrocallistes vastus *(Order Hexactinosida, Family Aphrocallistidae), compares it to previously published poriferan mtDNAs, and highlights two translational frameshifts, a phenomenon that is unique to the hexactinellids among reported lower metazoan mitochondrial genomes.

## Results and Discussion

### Gene content

**Figure 1:**
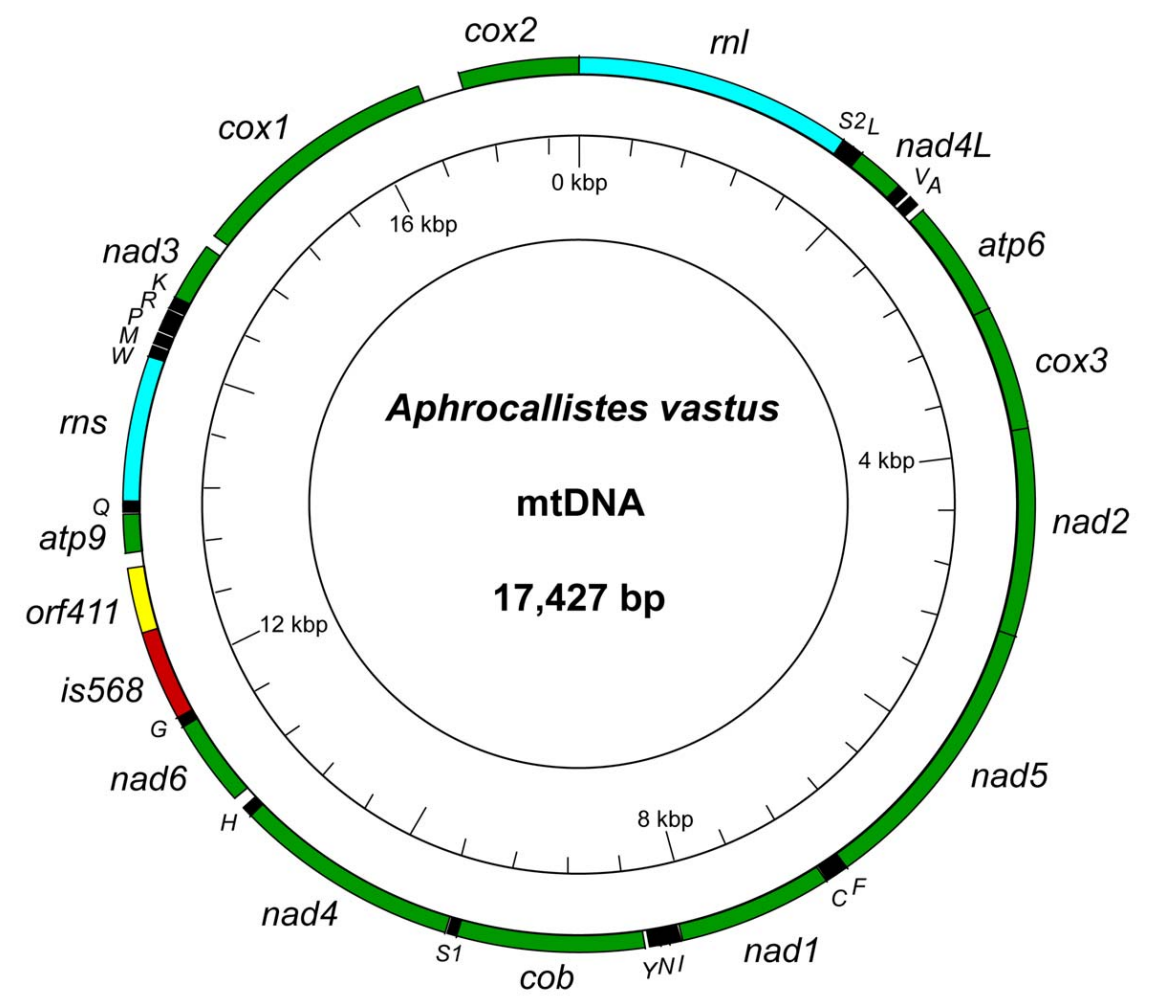
***Aphrocallistes vastus *mtDNA**. The mitochondrial genome of the hexactinellid *Aphrocallistes vastus *was determined to be a 17,427 bp circular molecule encoding 13 respiratory genes, two ribosomal RNAs and 18 tRNAs. Protein coding genes are represented in green, ribosomal RNAs in blue, and tRNAs with black boxes. The 411 bp unknown open reading frame (*orf411*) is shown in yellow, and a 568 bp intergenic space (*is568*) – a putative control region – is in red. Transfer RNAs are labeled with their one letter IUPAC amino acid abbreviation. All genes are encoded on the same strand.

**Figure 2:**
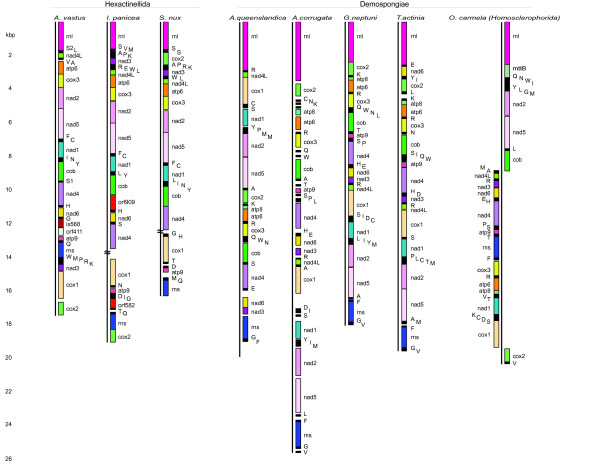
**Linearized genomes of all sequenced poriferan mtDNAs**. Side-by-side comparison of the mtDNAs of *A. vastus *and the previously published hexactinellid sponge mtDNAs reveal a large syntenic gene block: *atp6-cox3-nad2-nad5-trnF-trnC-nad1-trn(L, I, N, Y)-cob*. Variable gene arrangements among the hexactinellids include, but are not limited to, the order of *cox2-rnl*, the transposition of *nad3*, and the presence and location of *nad6*. Demosponges (excepting the homoscleromorph *O. carmela*) retain the syntenic block *cox2-atp8-atp6-cox3 *which is highly conserved throughout much of the animal kingdom, while the hexactinellids have lost *atp8 *and translocated *cox2*.

The complete mitochondrial genome of *Aphrocallistes vastus *was sequenced and shown to be a 17,427 base pair circular molecule encoding 13 proteins, 2 ribosomal RNA subunits, and 18 tRNAs [Genbank: EU000309] (Figure 1, Table 1). All genes were found to be coded on the same strand. The protein coding genes included 12 of the respiratory genes (*atp6, cob, cox1-3, nad1-4, 4L, 5, 6*) common to most animal mtDNA, as well as the ATP synthase F0 subunit9 (*atp9*) found in all published sponge mitochondrial genomes except for *Amphimedon *[13-16,19]. A 411 bp open reading frame (*orf411*) of unknown identity and function was located adjacent to the largest, 568 bp, intergenic space (*is568*), just downstream of the *nad4 *+ *trnH *+ *nad6 *+ *trnG *genes (Figures 1 and 2). *orf411 *does not display significant nucleotide or amino acid similarity to either of the unknown ORFs in the *I. panicea *mtDNA. *is568 *contains numerous direct repeats and may comprise a control region that includes the origin of replication. A putative control region was inferred in the mitochondrial genome of *Amphimedon *as well [13].

**Table 1:** *Aphrocallistes vastus *mtDNA gene content, size and overlap

	**Gene**	**Length (bp)**	**Overlap/length (bp)**
Protein coding	*atp6*	729	
	*atp9*	237	
	*cob*	1182	*trnR(ucu)*/28
	*cox1*	1569	
	*cox2*	741^T^	
	*cox3*	784*	*nad2*/42
	*nad1*	951	
	*nad2*	1401^T^	*cox3*/42 *nad5*/58
	*nad3*	363	
	*nad4*	1416	*trnH(gug)*/14
	*nad4L*	303	
	*nad5*	1881	*nad2*/58 *trnF(gaa)*/71^# ^*trnC(gca)*/7
	*nad6*	568*	*trnG(ucc)*/19
Ribosomal RNAs	*rnl*	1718**	
	*rns*	918**	
Transfer RNAs	*trnS1 (ucu) trnS2 (uga)*	64 67	*cob*/28
	*trnL (uag)*	66	
	*trnV (uac)*	70	
	*trnA (ugc)*	69	
	*trnF (gaa)*	72	*nad5*/71^#^
	*trnC (gca)*	64	*nad5*/7
	*trnI (gau)*	66	
	*trnN (guu)*	68	
	*trnY (gua)*	66	
	*trnR(ucg)*	72	*trnP(ugg)*/1
	*trnH (gug)*	68	*nad4*/14
	*trnG (ucc)*	65	*nad6*/19
	*trnQ (uug)*	70	
	*trnW (uca)*	69	
	*trnM (cau)*	72	
	*trnP (ugg)*	67	*trnR(ucg)*/1
	*trnK (uuu)*	69	
Missing tRNAs	*trnD, trnE, trnT*		

**t **Uses the TAG stop codon, while those without notation all use TAA.

^# ^The predicted *trnF *gene lies entirely within the *nad5 *ORF.

*The gene length includes both reading frames inferred to constitute the protein.

**The start and stop positions of the ribosomal RNAs were predicted based on conserved multiple alignment positions and the borders with neighboring genes.

The beginnings and ends of the small and large rRNA subunits, 918 bp and 1718 bp, respectively, were approximated from alignment data as these genes retained little sequence similarity to the rRNAs from sequenced demosponge mtDNAs, or with those in the non-redundant BLAST database. Thus the complete rRNA sequences remain to be confirmed experimentally. The *A. vastus *rRNAs were similar to the predicted rRNAs of *I. panicea *and *S. nux*, and the *rnl *sequence was highly similar to the partial 16S rRNA from the hexactinellid *Heterochone calyx *[Genbank: AM183122], indicating that these genes are similar within the hexactinellids but divergent from those of other sponge classes. Whereas the first three published sponge mtDNAs, all of closely related demosponges, presented a picture of highly similar, conserved molecules [15,19], additional demosponge and hexactinellid mtDNAs have since revealed that some sponge mtDNAs have unknown ORFs, genes on the complement strand (as in *Oscarella carmela*), and divergent gene sequences [13,14,16].

**Figure 3:**
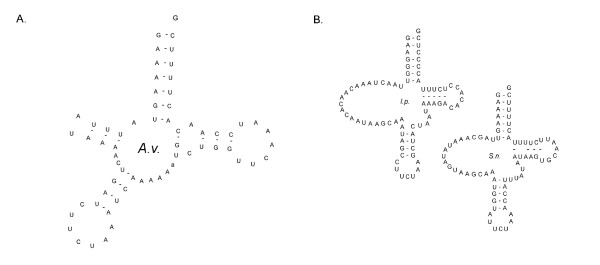
**The structures of hexactinellid mtDNA encoded *trnS(ucu)***. (A) The structure of *trnS(ucu) *from *A. vastus *(redrawn from the tRNAscan-SE prediction) is peculiar, but unique among the hexactinellids. (B) The *trnS(ucu) *genes of *I. panicea *and *S. nux *(redrawn from Haen *et al.*, 2007) share an unusual structure similar to that found in some bilaterians.

The tRNA complement of *A. vastus*, 18 genes predicted by tRNAscan-SE and confirmed by BLAST similarity searches [20,21], was much reduced compared to that of demosponges, and even sparser than the other reported hexactinellids, which had 22 and 20 tRNAs [14]. It included 2 isoacceptors for serine, *trn*^*Ser*^*(ucu) *and *trn*^*Ser*^*(uga)*, while three tRNAs are missing entirely: *trnD*, *trnE*, and *trnT *(Table 1). A reduction in mitochondrial encoded tRNAs, therefore, represents a polyphyletic characteristic between lower metazoan groups, found most extremely among cnidarians which have lost nearly all of their mtDNA encoded tRNAs [22]. The structure of the *A. vastus trnS1*, predicted by tRNAscan-SE, reveals a DHU arm with a uniquely truncated D-loop, a feature distinct from the novel *trnS *genes in *I. panicea *and *S. nux*, which have a loop in place of a DHU arm, reminiscent of bilaterian tRNAs (Figure 3). The remaining *A. vastus *tRNAs display traits similar to those of the other hexactinellids – namely polymorphic DHU and TψC arms, and loss of the canonical guanine bases in the DHU loop. Numerous tRNAs (*trnA*, *trnR*, *trnQ*, *trnG*, *trnI*, *trnK*, *trnF*, *trnP*, and *trnY*) have mismatches in their acceptor arm, as well as in their anticodon arm (*trnS2*, *trnI*, and *trnQ*) and T-arm (*trnR*, *trnM*, and *trnS1*) (Additional file 1), a common feature in higher animal mitochondrial tRNAs that is corrected through RNA editing [23].

### Genome organization, nucleotide composition and codon usage

The organization of the *A. vastus *mtDNA was quite compact, typical of sponges and most other animals. Nearly 72% of the mtDNA was predicted to encode proteins (including *orf411*), 22% to encode ribosomal and transfer RNAs, while only 6% was non-coding intergenic space. Almost 52% of the intergenic space was comprised of the predicted control region, *is568 *(Figure 1). Furthermore, there were eight examples of overlapping genes, including one remarkable instance in which the *trnF *gene was found entirely contained within the *nad5 *open reading frame (Table 1). Four of the eight gene overlaps involve the *nad2-nad5 *gene block, indicating that this region has experienced a higher rate of compaction than other parts of the mtDNA.

Nucleotide composition of *A. vastus *was found to be similar to that of the reported hexactinellids in terms of A-T richness and nucleotide skew, as was its codon usage. The *A. vastus *mtDNA was calculated to be 70.7% A+T, nearly identical to that of *S. nux *(70.4%). The AT skew (calculated as (A-T)/(A+T)) and GC skew (calculated as (G-C)/(G+C)) for the coding strand of *A. vastus *were 0.18 and -0.28, again nearly identical to those of *S. nux *(0.19 and -0.28). While the skew metrics were quite similar among the hexactinellids, they ran counter to the compositional preferences of the demosponges, which display negative AT and positive GC skews. All three known hexactinellids share a reassignment of AGR codons from ariginine to serine (see Haen *et al.*, 2007 for a detailed analysis). This change is not seen in the demosponges or in other lower metazoan taxa, but rather is known only from select bilaterian groups. The mtDNA of *A. vastus*, like the other glass sponges, encodes only a *trnI(gau) *but heavily favors the AUA codon to code for isoleucine (Table 2). It should be noted that the *A. vastus *mtDNA was found to lack several tRNAs, specifically for the amino acids aspartic acid, glutamic acid, and threonine, and thus must import some nuclear-derived tRNAs to complete translation.

**Table 2:** Exceptional mtDNA codon usage

		**Hexactinellida**			**Demospongiae**				
		
		***A.v.***	***I.p.***	***S.n.***	***A.q.***	***A.c.***	***G.n.***	***T.a.***	***O.c.***
AA	codon								
Ile	AUA	458	628	433	221	205	218	284	278
	AUU	128	69	161	95	222	207	209	160
	AUC	91	107	86	46	45	18	3	38
Arg	**AGG**				32	17	15	18	12
	**AGA**				42	35	55	49	54
	CGG	1	2	0	23	14	4	8	3
	CGA	44	56	51	25	16	21	11	21
	CGU	5	4	3	1	13	5	12	10
	CGC	4	13	4	2	4	0	0	0
Ser	**AGG**	3	10	7					
	**AGA**	187	221	175					
	AGU	21	16	9	65	74	79	113	68
	AGC	15	34	14	25	23	15	3	24
	UCG	0	3	3	48	37	25	17	10
	UCA	84	71	85	52	69	67	92	80
	UCU	56	41	38	49	94	106	99	100
	UCC	44	67	43	36	21	16	3	12
Trp	UGG	0	0	3	62	22	20	31	14
	UGA	72	97	76	39	68	62	58	75

For each sponge species with a published mtDNA, the number of occurrences in protein coding sequence is given of codons with exceptional usage (*A.v., Aphrocallistes vastus; I.p., Iphiteon panicea; S.n., Sympagella nux; A.q. Amphimedon queenslandica; A.c., Axinella corrugata; G.n., Geodia neptuni; T.a., Tethya actinia; O.c., Oscarella carmela*). The hexactinellid sponges strongly favor Ile(AUA) despite coding for a tRNA with (*gau*) specificity. AGR codons (in bold) are reassigned from arginine to serine in the glass sponges. The hexactinellids rarely if ever employ the Trp(UGG) codon for translation, but rather seem to use it as a signal for programmed frameshifting.

### Regions of synteny and unique transpositions among the hexactinellids

The largest block of genes shared between *A. vastus *and the previously published hexactinellid sponge mtDNAs consisted of *atp6-cox3-nad2-nad5-trnF-trnC-nad1-trn(L, I, N, Y)-cob*. *Aphrocallistes *contained *trnI, N*, and *Y *between *nad1 *and *cob*, while *I. panicea *had *trnL *and *trnY*, and *S. nux *retained the entire complement *trnL, I, N*, and *Y*. This region of synteny spanned 7,127 bp of the *A. vastus *mtDNA, or 41% of the genome (Figures 1 and 2). *Aphrocallistes *and *I. panicea *shared the *cox2-rnl *gene border, while in *S. nux *these genes were shuffled to *rnl-cox2*. With respect to hexactinellids, *nad3 *has transposed in the *A. vastus *mtDNA, adjacent to *cox1*, whereas it is adjacent to tRNAs and *nad4L *in *I. panicea *and *S. nux*. In *A. vastus *and *I. panicea*, *nad4 *and *nad6 *are adjacent but found in the opposite order. While *nad6 *has not been found in the sequenced portion of the *S. nux *mtDNA, the gap in the genome lies upstream of *nad4*. If *nad6 *is found in this gap upon completion of the *S. nux *mtDNA sequence, *S. nux *would share the *nad4*-*nad6 *boundary with *A. vastus *(Figure 2).

The mtDNA gene segment *cox2-atp8-atp6-cox3 *is a highly conserved syntenic region found in arthropods, echinoderms, chordates, and other higher animal groups [10]. In the choanoflagellate *Monosiga brevicollis *the order of these four genes relative to one another is conserved, though the genes are not found as a contiguous block [10]. Placozoan mtDNA does not contain this synteny [12,17], but it is present in all known demosponges except *O. carmela *(Figure 2) [13,15,16,19]. The synteny is also found in several described mtDNAs of octocorallians [22]. Demosponges appear to be the most basal animal group to retain this plesiomorphy, as the hexactinellids have lost *atp8 *and translocated *cox2 *(Figure 2). All sponge mtDNAs sequenced to date revealed a highly conserved synteny between the *nad2-nad5 *gene block. Meanwhile, the *cox1-tRNA(s)-nad1 *region shared among the demosponges, including *O. carmela*, was not found in any of the hexactinellid mtDNAs (Figure 2). Recall that *nad1 *was part of the large hexactinellid-specific synteny described above.

### Translational frameshifting in *cox3 *and *nad6*

**Figure 4:**
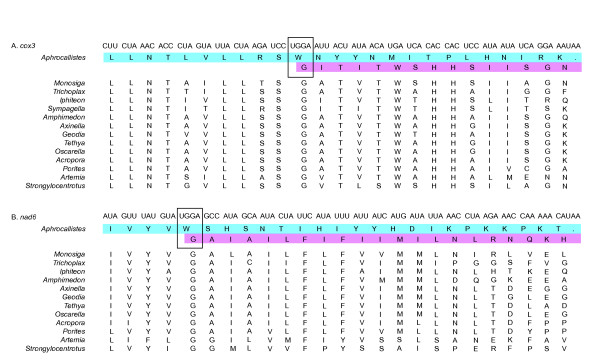
**Frameshifts in the *cox3 *and *nad6 *genes**. The genes *cox3 *(A) and *nad6 *(B) in the *A. vastus *mtDNA both contain +1 frameshifts at the rare tryptophan codon UGG. After the UGG, the amino acid sequence of first frame (blue shading) becomes degenerate and reaches a premature stop codon. Multiple alignment of these genes from closely- and distantly-related animal taxa reveal conservation of the amino acid sequence in the +1 frame (pink shading), achieved by shifting from the codon UGG to GGA (boxes).

*Aphrocallistes cox3 *and *nad6 *genes were found to contain a +1 translational frameshift at amino acid position 140 and 58, respectively. In both cases, the predicted amino acid sequence encoded by the *cox3 *and *nad6 *open reading frames prior to this frameshift was highly similar to the N-terminal portion of diverse metazoan homologs. Coincident with the +1 frameshift in these genes, the predicted amino acid sequence in frame 2 encoded the remaining C-terminal portion of these proteins based on multiple alignments to poriferan, diploblast and bilaterian homologs (Figure 4). The frameshift occurred precisely at the codon UGG (tryptophan) for both genes. The UGG codon has been suggested to play a role in translational frameshifting in the other reported hexactinellids, albeit in different genes than in *A. vastus *[14]. The amino acid tryptophan was found in 72 other positions in the predicted *A. vastus *mtDNA proteome, each time coded for by UGA (Table 2). The only two instances of the UGG codon appeared precisely at the frameshifts in *cox3 *and *nad6*. Moreover, the frame 1 tryptophan is replaced by a highly conserved frame 2 glycine that is found in all taxa in the alignment. Numerous other widely shared residues followed the glycine in frame 2 (Figure 4).

One possibility is that the frameshift goes uncorrected and that functional proteins are encoded by nuclear copies of the genes. If this were the case, one would expect these mitochondrial genes to have diverged under relaxed selective pressure. The multiple alignment shows, however, that the amino acid sequences on both sides of the frameshift have retained strong similarity to closely and distantly related homologs (Figure 4). A second possibility is the frameshifts were corrected by RNA editing, as is commonly seen in the mtDNA of plants, fungi, and protists [24-27]. To test this possibility, a randomly primed cDNA pool was screened by PCR with *cox3 *and *nad6 *gene specific primers flanking the UGG codon. The amplified cDNA fragments from several independent PCR reactions were sequenced. The results (unpublished data) showed that all cDNA products contained the frameshift, (a) demonstrating that the mitochondrial genes are expressed, and (b) ruling out the possibility of an RNA editing mechanism to restore the *cox3 *and *nad6 *reading frames.

Yet another possibility is that the frameshift was corrected by translational frameshifting. A mechanism for +1 programmed translational frameshifting has been described in *Saccharomyces cerevisaie *[28-31] and reported in disparate bilaterian animal groups [32,33]. The phenomenon occurs in three steps: first, the ribosomal P site-bound peptidyl-tRNA forms a "weak wobble interaction" with the gene transcript; second, translation halts because the next required tRNA is so rare that its codon is not readily recognized; and lastly, an abundant aminoacyl-tRNA of the +1 codon forces the ribosomal complex to shift frames [30]. This scenario can be readily applied to the *A. vastus cox3 *and *nad6 *genes. We hypothesize that translation pauses due to poor recognition of the highly unusual UGG tryptophan codon, allowing an abundant tRNA^*Gly*^(GGA) to induce the +1 frameshift. This hypothesis begs many questions. For example: How efficient is the mechanism of frameshift correction? If the frameshift lowers the translation efficiency of the affected genes, why has selection allowed a frameshift to persist? Has this mechanism evolved convergently in each disparate taxa, or is it a shared but rarely employed tool available to any animal whenever a frameshift becomes fixed in the mitochondrial genome?

## Conclusion

The few sequenced hexactinellid sponge mtDNAs contain a suite of shared traits, including the loss of the gene *atp8*, retention of the gene *atp9*, highly divergent ribosomal RNAs, and reduction of their tRNA complements. Hexactinellid nucleotide composition is distinct from that of demosponges, favoring adenine over thymine and cytosine over guanine on the coding strand. The hexactinellid mtDNAs share a large region of synteny spanning the *atp6 *to *cob *genes, but a syntenty putatively ancestral to the Metazoa, *cox2-atp8-atp6-cox3*, is not retained. Perhaps the most unique feature of hexactinellid mitochondrial genomes is the predicted +1 programmed translational frameshifting initiated by ribosomal pausing at the extremely rare UGG (tryptophan) codon. Future mtDNA sampling will reveal whether this phenomenon is common to yet more glass sponges or the other sponge classes.

## Methods

### Tissue preparation and DNA purification

The hexactinellid sponge *Aphrocallistes vastus *was collected using the manipulator arm of the remote operated vehicle ROPOS (Remote Operated Platform for Ocean Science; ropos.com) at San Jose Islets, Barkley Sound, Canada, and transferred without removal from seawater to tanks at the Bamfield Marine Sciences Centre. Tissue was cut into small pieces, and dissociated through Nitex mesh into cold seawater and allowed to reaggregate over 2 days to eliminate possible contamination by other taxa and to facilitate DNA preparation. Aggregates were cleaned twice daily with sea water and frozen directly at -80°C. The tissue was thawed and lysed simultaneously in 8 M urea buffer, incubated at 65°C for 20 min, and total DNA was prepared by phenol chloroform extraction and precipitation in isopropanol [34].

### mtDNA PCR amplification, cloning and sequencing

A genome walker library was constructed from *A. vastus *genomic DNA using the GenomeWalker Kit (BD Biosciences) and restriction enzymes DraI, EcoRV, PvuII, SmaI, and StuI. This library was screened by PCR with universal 16S primers and adapter primers AP1 and nested AP2 to amplify a fragment of *rnl*. The initial contig was generated with primer P1313 (caattcaacatcgaggtcgcaaaca) and AP1(gtaatacgactcactatagggc). Genome-walking was continued until a 12 kb contig had been partially sequenced. Long-range primers designed against the ends of this contig were successful in amplifying the entire contig using TAKARA LA-Taq. This product was purified using the QiaQuick Gel Extraction kit (Qiagen), sheared by sonication, and end-repaired with the DNA Terminator kit (Lucigen). Two to four kilobase fragments were size-selected by gel electrophoresis, blunt-end cloned into the pSmart LC-Kan vector (Lucigen), and transformed into *E. cloni *Supreme cells (Lucigen) by electroporation. Colonies were screened by PCR for the presence of inserts using flanking vector primers SL1 and SR2 (Lucigen). Forty-eight PCR products were purified by poly-ethylene glycol-NaCl (PEG:NaCl) [35] and sequenced by BigDye^® ^Terminator v3.1 cycle sequencing on ABI PRISM^® ^3700 DNA Analyzers (Applied Biosystems, Inc.) at the W.M. Keck facility at Yale University, New Haven, CT. Outward facing primers from the 12-kb contig were designed, and a 5.5 kb fragment was amplified also using TAKARA LA-Taq following manufacturers instructions. This product was gel-purified with the Qiagen kit, ligated into the PCR 2.1 Topo vector (Invitrogen) and transformed into Top10 cells. Four clones were recovered, grown overnight in LB and mini-prepped using Qiagen Qiaquick columns. All four clones were sequenced by primer walking.

### Sequence assembly and annotation

Sequences were assembled from chromatography data using the Phred Phrap Consed software package, release 15.0 [36,37]. Regions of lower quality data were sequenced by direct PCR on genomic DNA, or additional sequencing of select gap spanning clones. The complete contig had a minimum of 2× coverage with high phred values (40 or greater) at each position. The suite of tRNA genes were identified by tRNAscan-SE [21] using the program's default parameters for organellar DNA and the Mold and Protozoan mitochondrial translation code. BLASTN searches querying all published sponge mtDNA tRNAs against the *A. vastus *genome did not identify any additional tRNAs, nor did manual searching for missing anticodon-loop motifs. Protein-coding and ribosomal RNA genes were initially identified using the program DOGMA [38], and then aligned by Blast2 [39] and ClustalW [40] with the mtDNA genes annotated in GenBank.

### RNA extraction and cDNA preparation

One ml of frozen cell aggregate was pulverized under liquid nitrogen. Total RNA was extracted from half of the resulting ground tissue using the Illustra RNAspin Mini kit (GE Healthcare). The total RNA was treated with Dnase I for 1 hour, then cleaned by phenol chloroform extraction, precipitated in cold 100% ethanol, and resuspended in DEPC-dH_2_O with 1 μl RNase Inhibitor (Roche). 5 μg of RNA was reverse transcribed with Invitrogen SuperscriptII reverse transcriptase and random oligos, and the resulting cDNA treated with RNase H. As a control for subsequent PCRs, a sample of RNA was processed in parallel, receiving identical treatment except without reverse transcriptase enzyme ("no-RT control"). The cDNA and no-RT control were used as PCR templates with primers pairs P2775 (agcagaacaaagaccatgacc) and P2964 (tggaatcctgtggctacaaagaaag) for *cox3 *and P3182 (aacatcttcaagaagaacaatcaatagag) and P2962 (catggttattatggtgcgttggatt) for *nad6*, using Qiagen PCR reagents and manufacturer's instructions. PCR products, amplified from the cDNA template alone, were cleaned with the QiaQuick PCR Purification kit (Qiagen) and sequenced directly using the above primers.

## List of Abbreviations

mitochondrial genome (mtDNA), ATP synthase F0 subunit # (*atp6, 8, 9*), apocytochrome b (*cob*), cytochrome c oxidase # (*cox1-3*), NADH dehydrogenase subunit # (*nad1-6, 4L*), small ribosomal RNA (*rns*), large ribosomal RNA (*rnl*), open reading frame (ORF), intergenic space (is)

## Authors' contributions

RDR was primarily responsible for the genome assembly, annotation, and comparative analysis, as well as the cDNA pool construction and screening, and manuscript preparation. EAS performed the bulk of the genome amplification, cloning and sequencing, and contributed to the genome assembly, annotation, and project conception. MAM isolated the total DNA, contributed significantly to the genome amplification, cloning and sequencing, and provided extensive technical support on other aspects of the project. SPL performed the organism collection, tissue cleaning, dissociation and reaggregation and provided the cellular material for DNA isolation. SLD was principal investigator, responsible for conception, design and advising at every stage of the project, as well as overseeing manuscript preparation. All authors have read and approved the final manuscript.

## Supplementary Material

Additional file 1 ***A. vastus *mtDNA tRNA structures**. Structure diagrams of all 18 *A. vastus *mtDNA encoded tRNAs predicted by tRNAscan-SE. Click here for file
