# Supplementary material for: The mitochondrial genome of the hexactinellid sponge Aphrocallistes vastus: Evidence for programmed translational frameshifting
Source: BMC Genomics. 2008 Jan 23;9:33. doi: 10.1186/1471-2164-9-33 (PMC2267718; doi:10.1186/1471-2164-9-33)

Alanine (A)

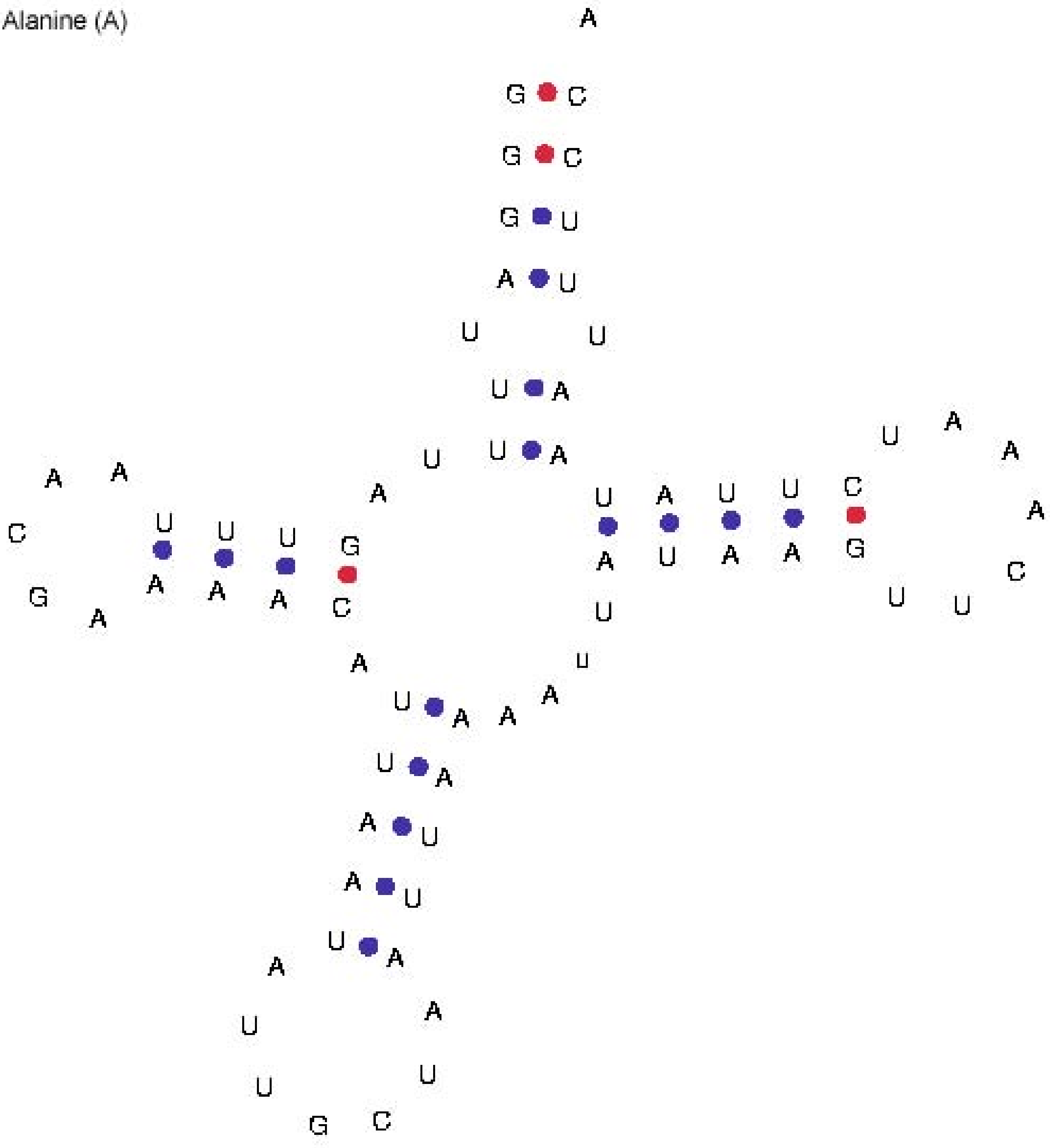

Arginine (R)

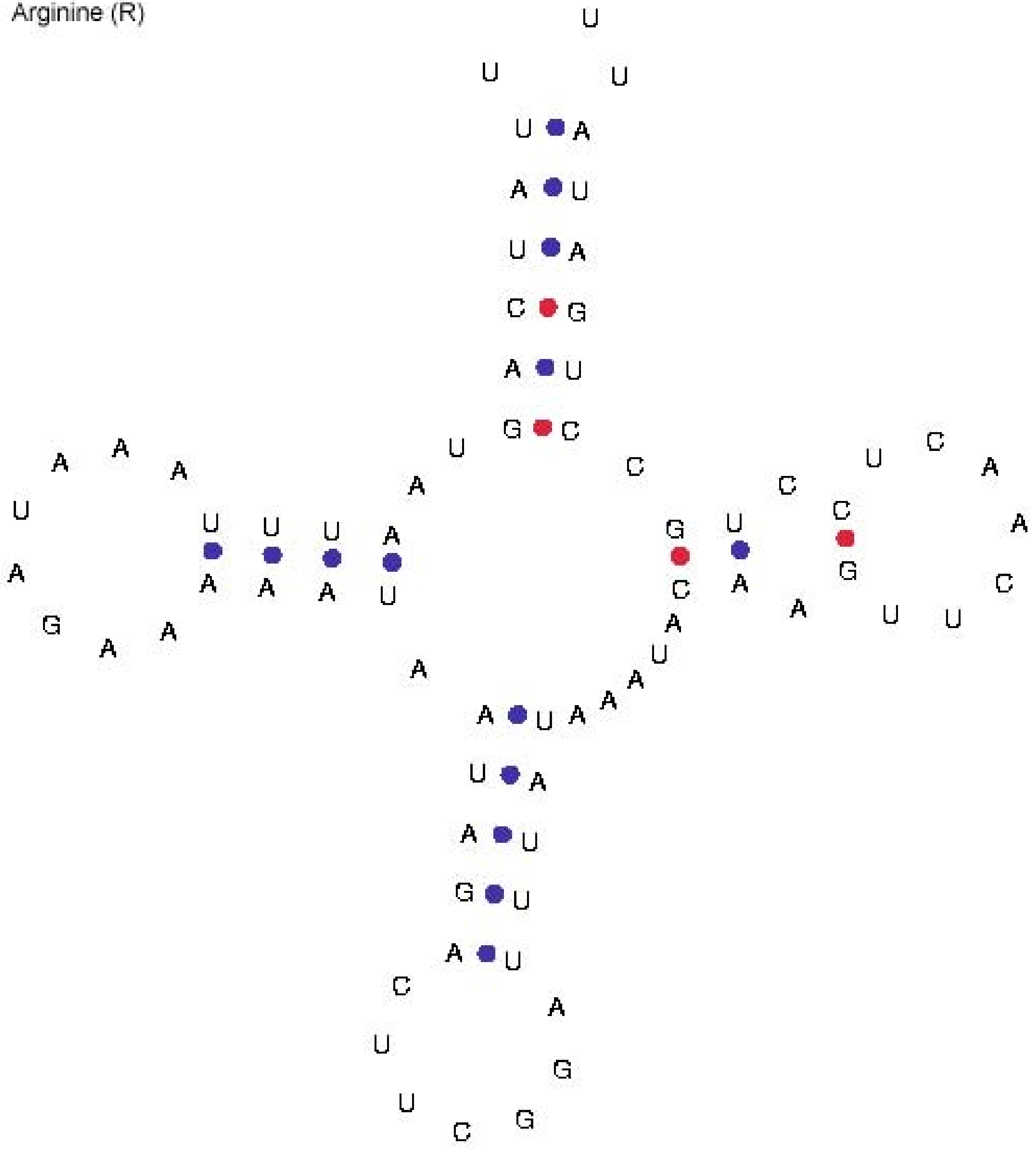

Asparagine (N)

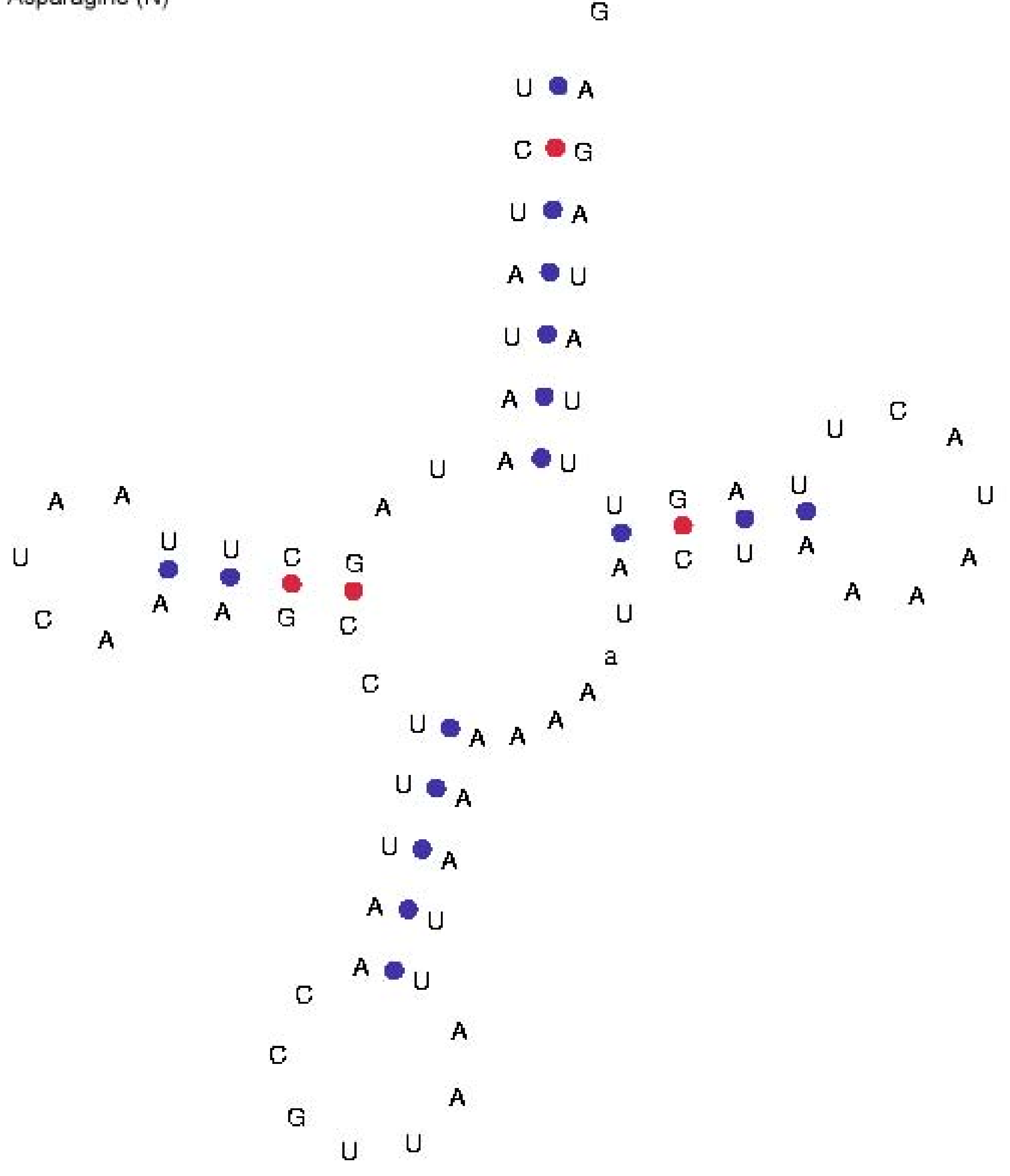

Cysteine (C)

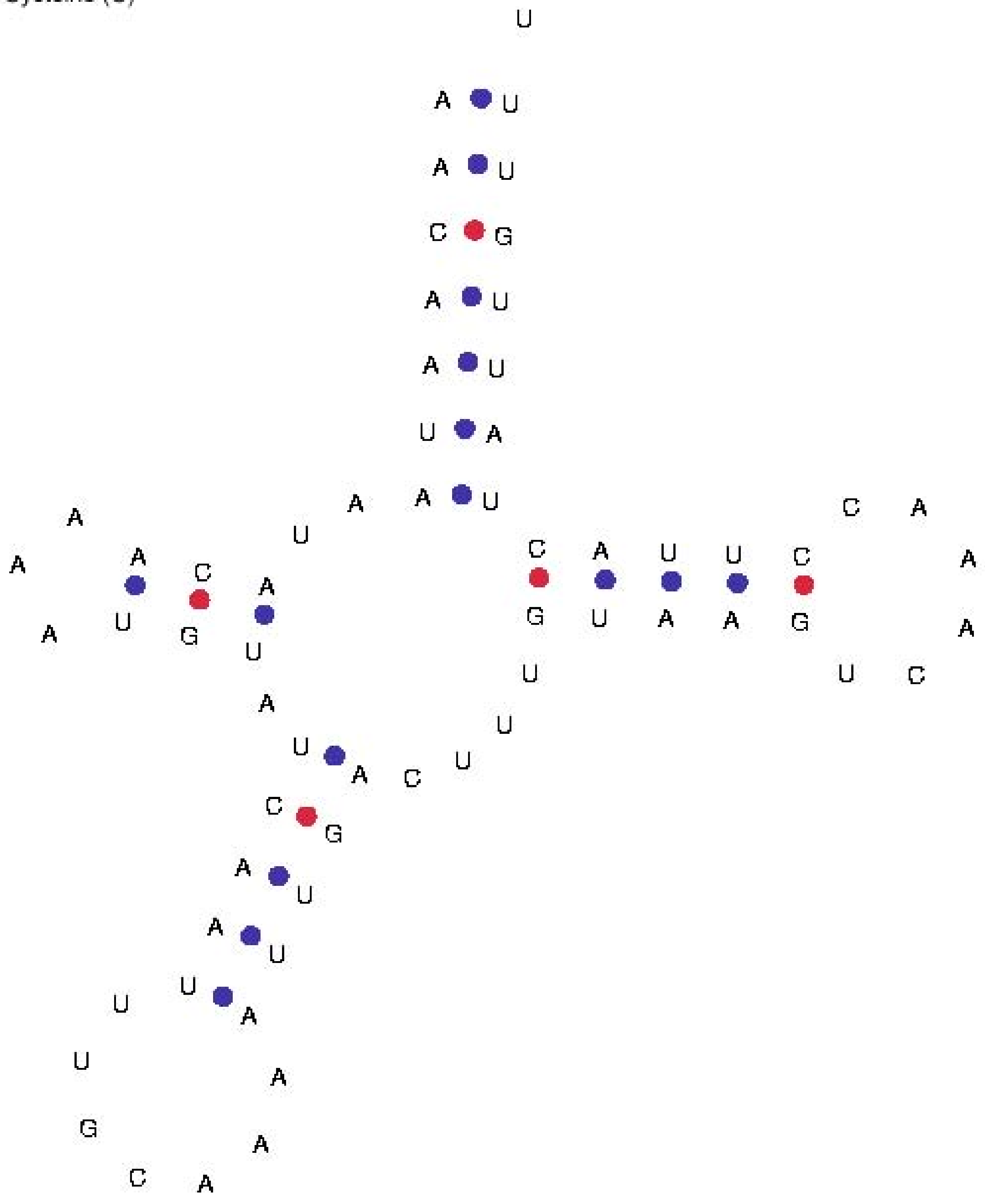
U
A U
A U
C G
A U
A U
U A
A A U
C A
A
U
C A U U C
A A
A
C
A
G U A A G
A U G
U
U C
A
U
U
U
A C
C G
A U
A U
U U A
U A
G A
C A

Glutamine (Q)

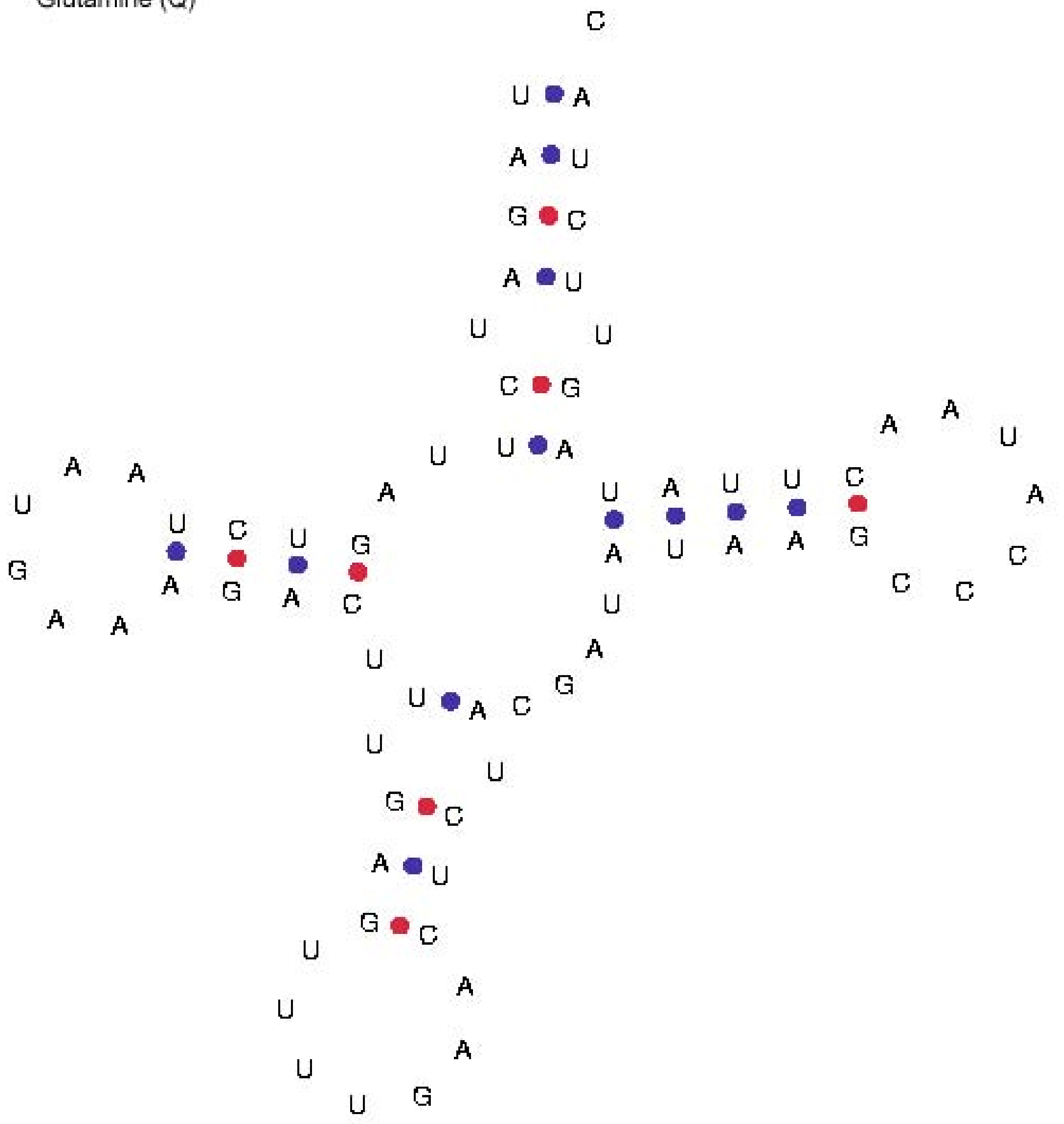

Glycine (G)

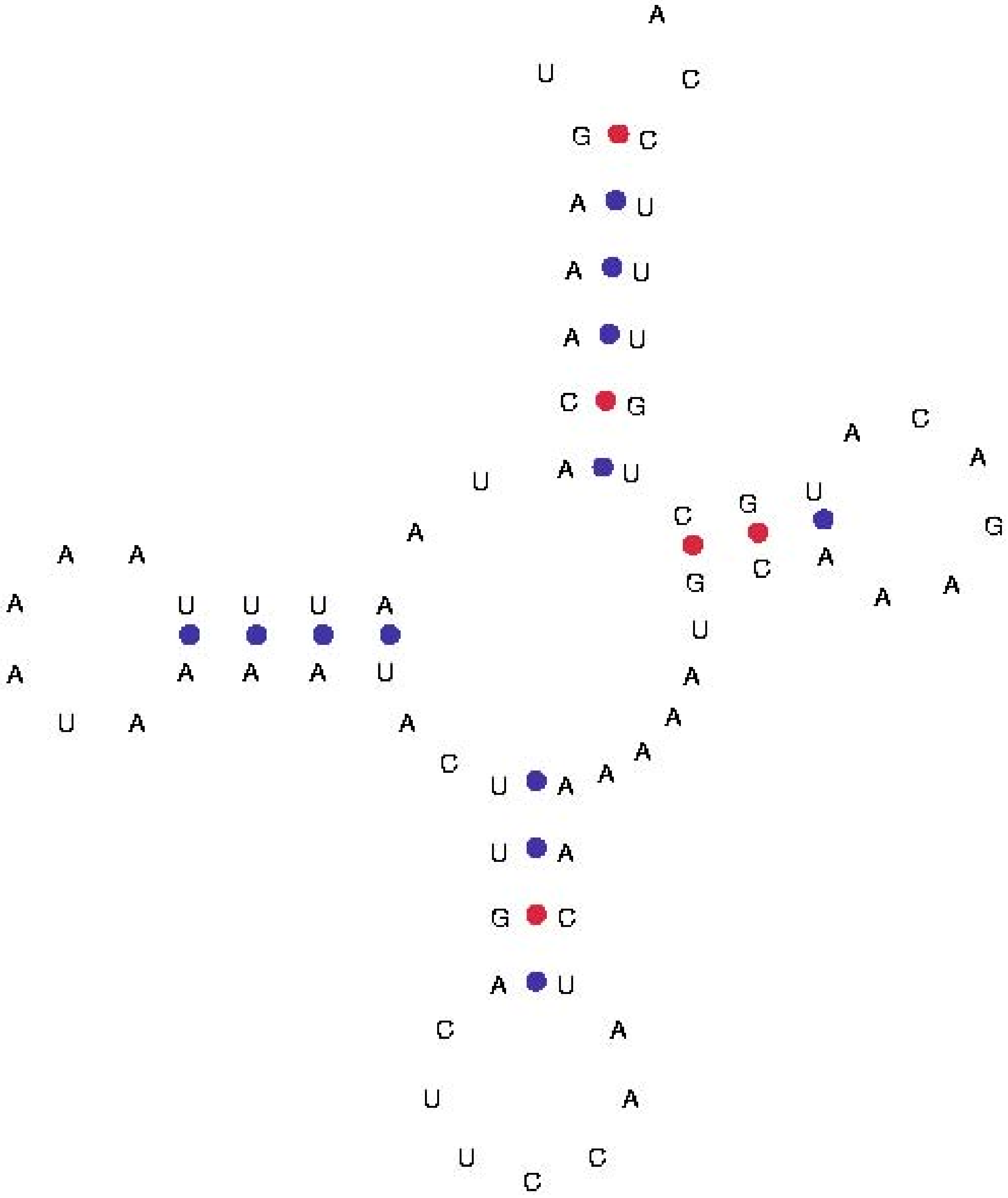

Histidine (H)

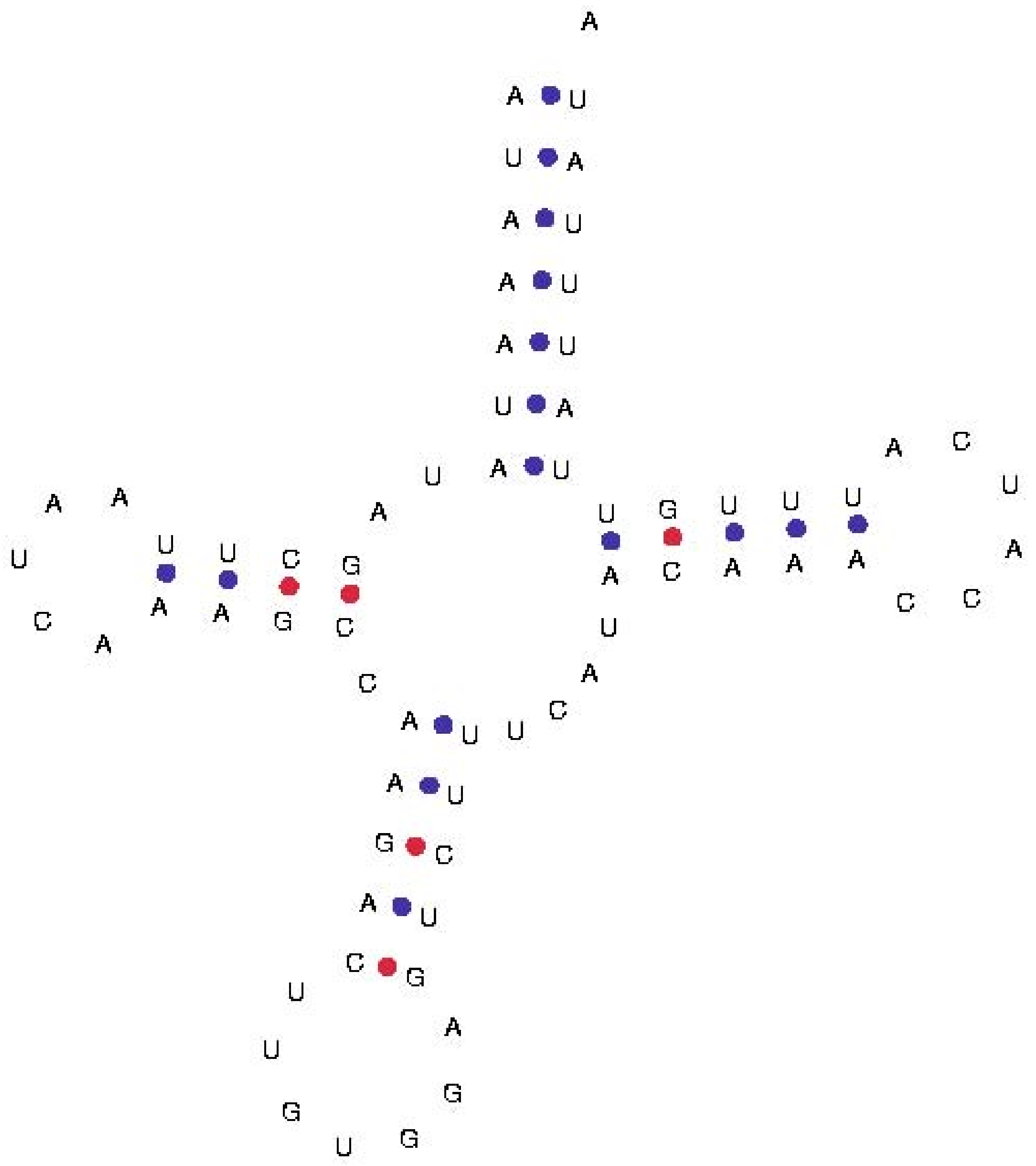

Isoleucine (I)

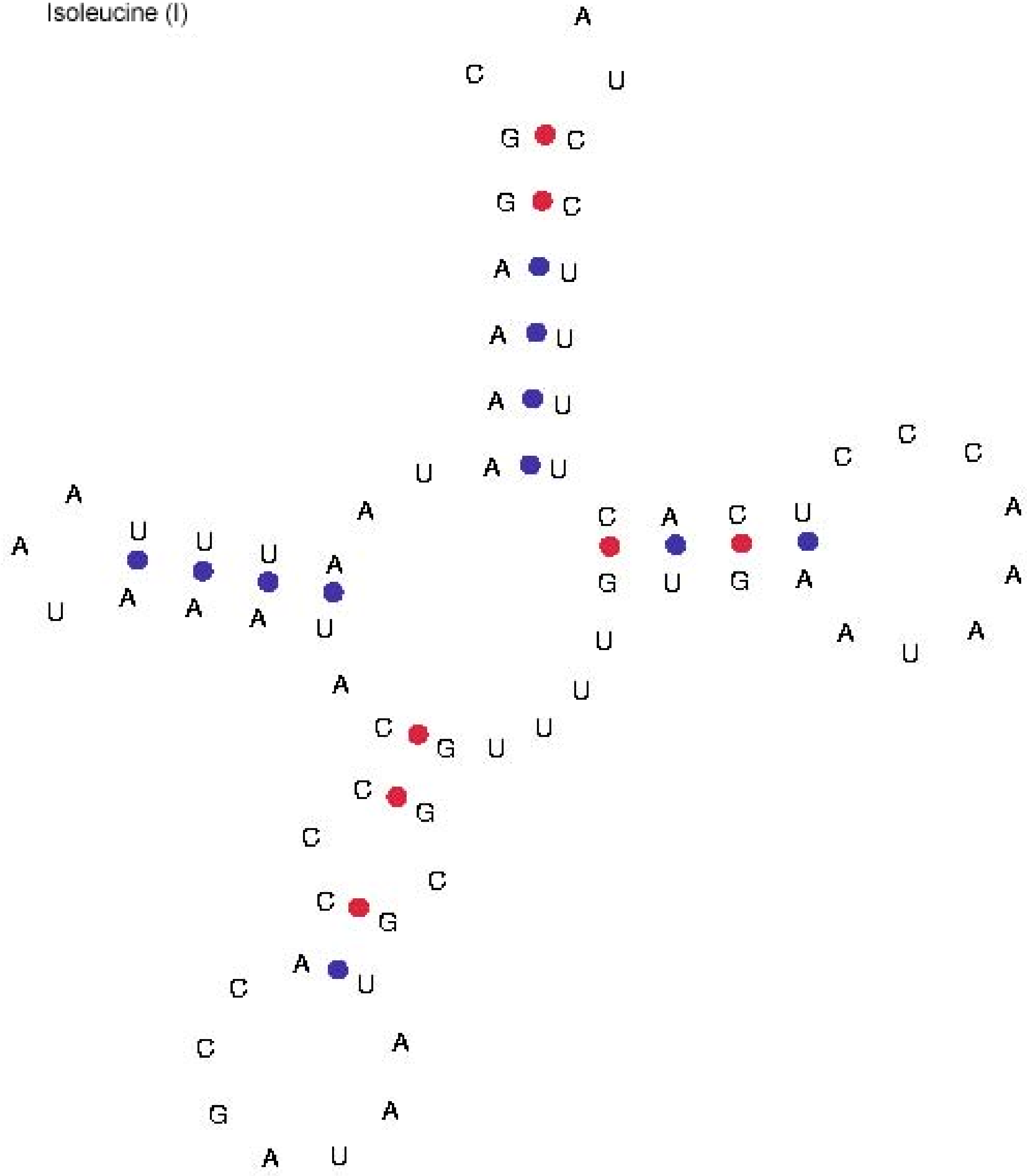

Leucine (L)

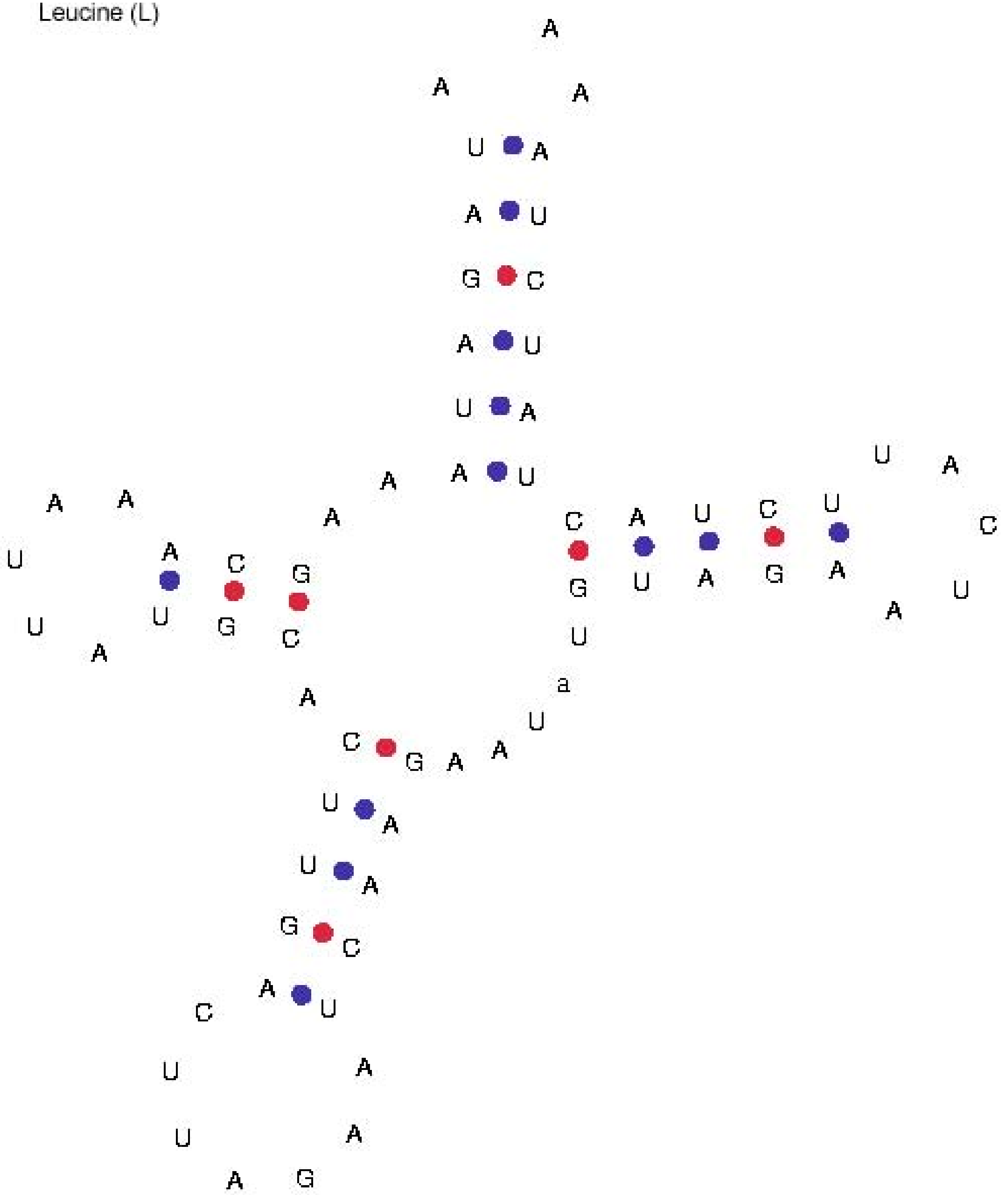

A
A A
U A
A U
G C
A U
U A
A A U U A
A A A C A U C U C
U A C G G U A G A U A
U U G C U
A a A
U C A G A
U A
U A
G C
A C U
U A
U A
A G

Lysine (K)

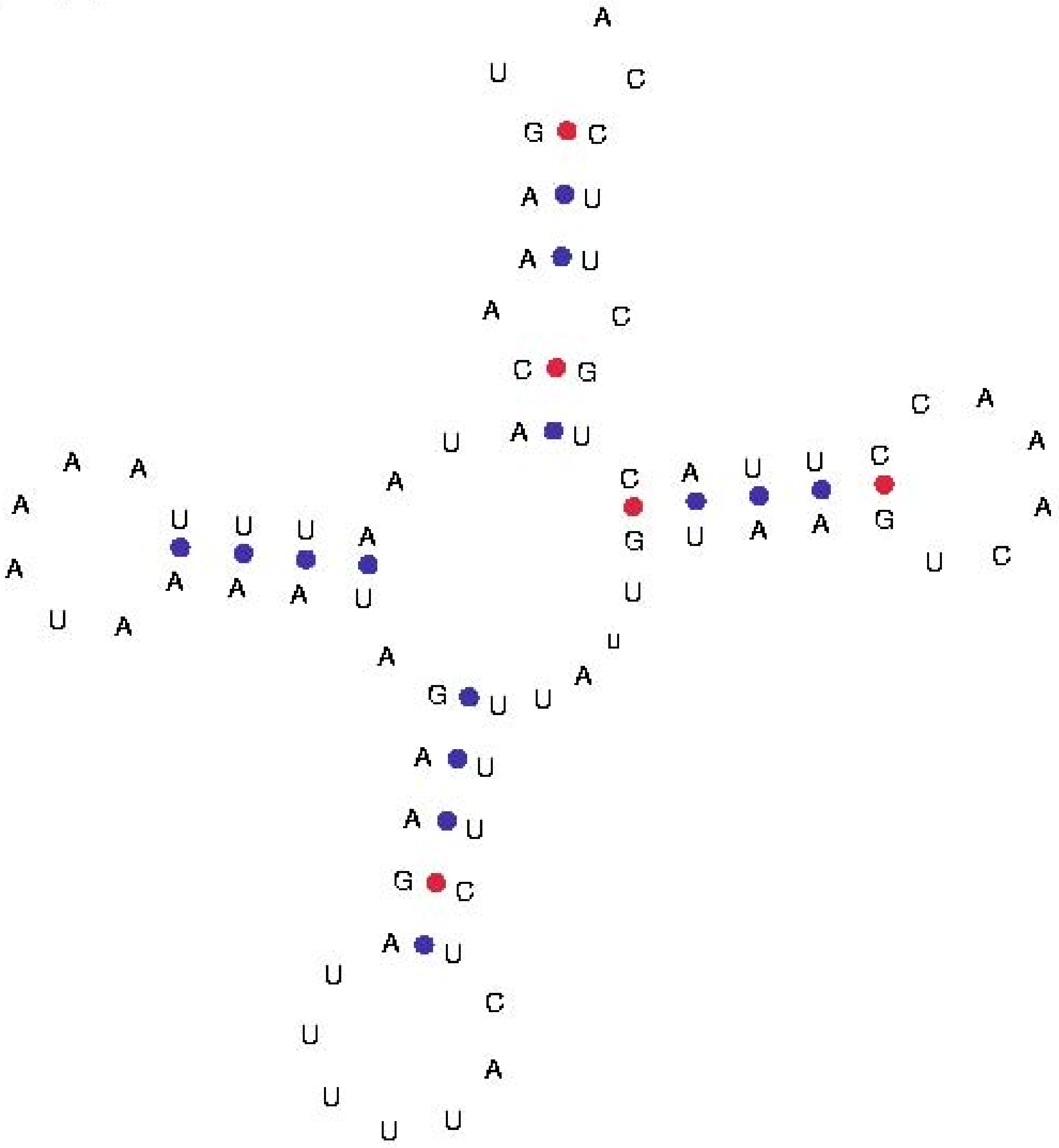

Methionine (M)

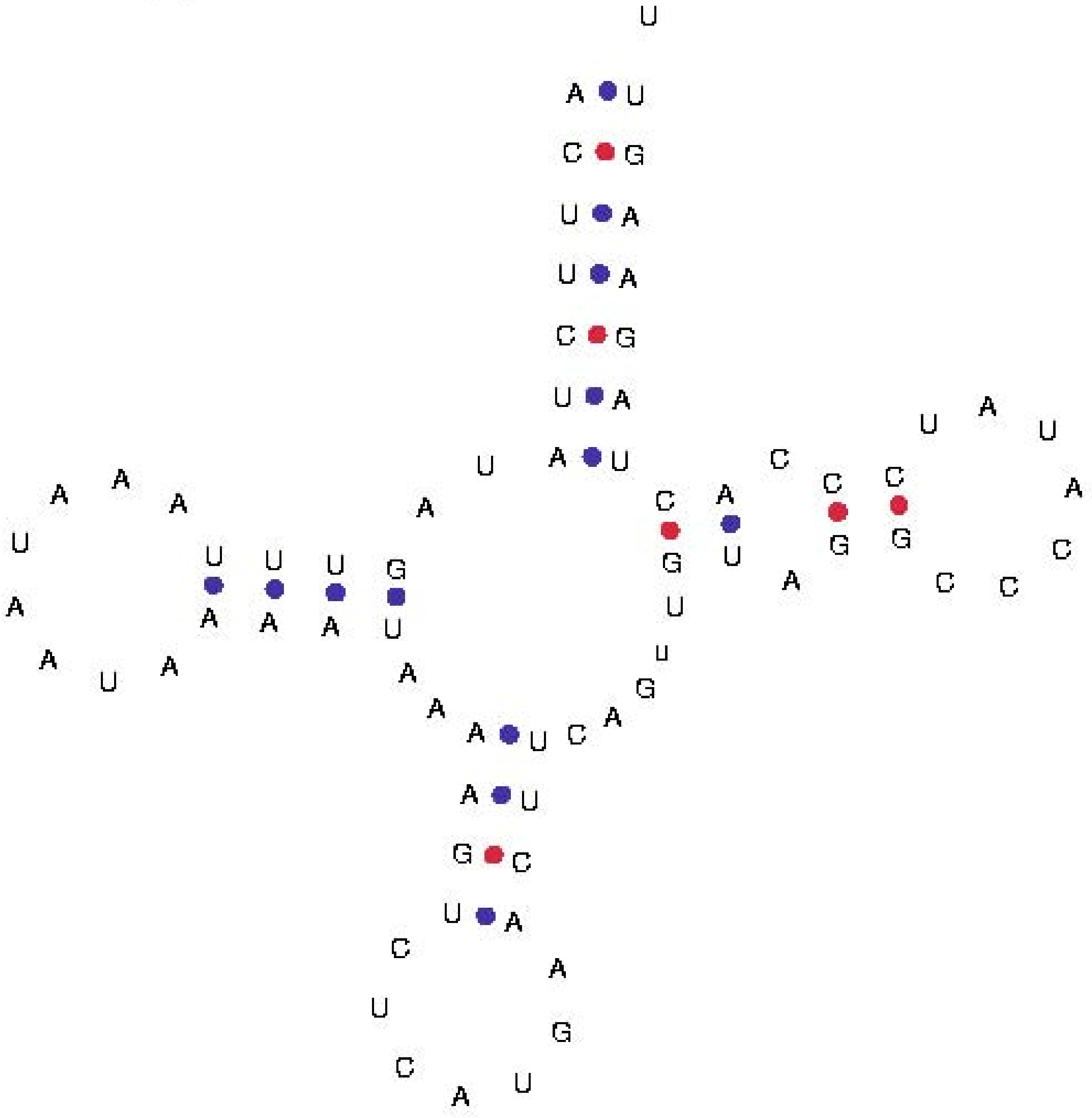

Phenylalanine (F)

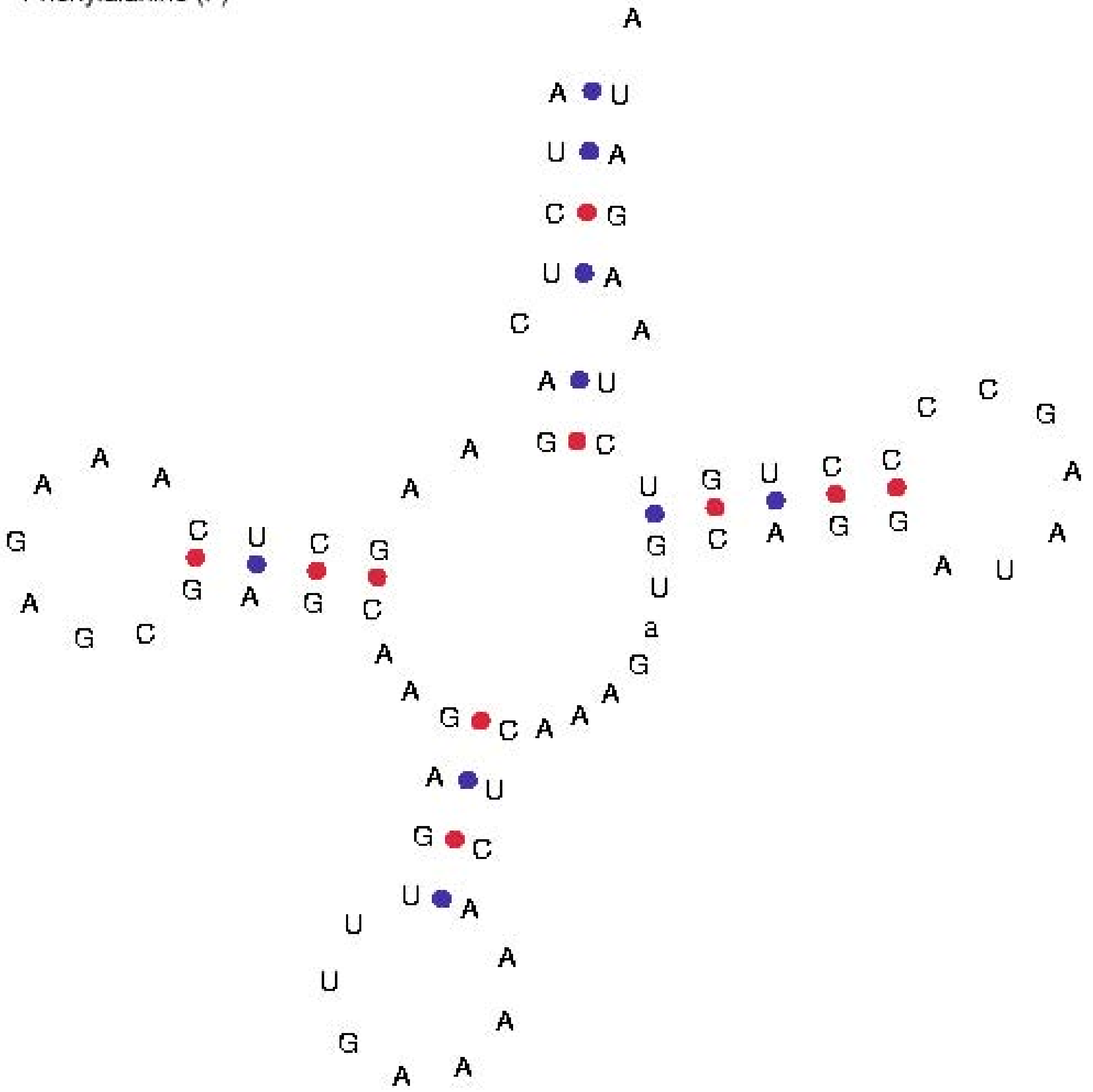

Proline (P)

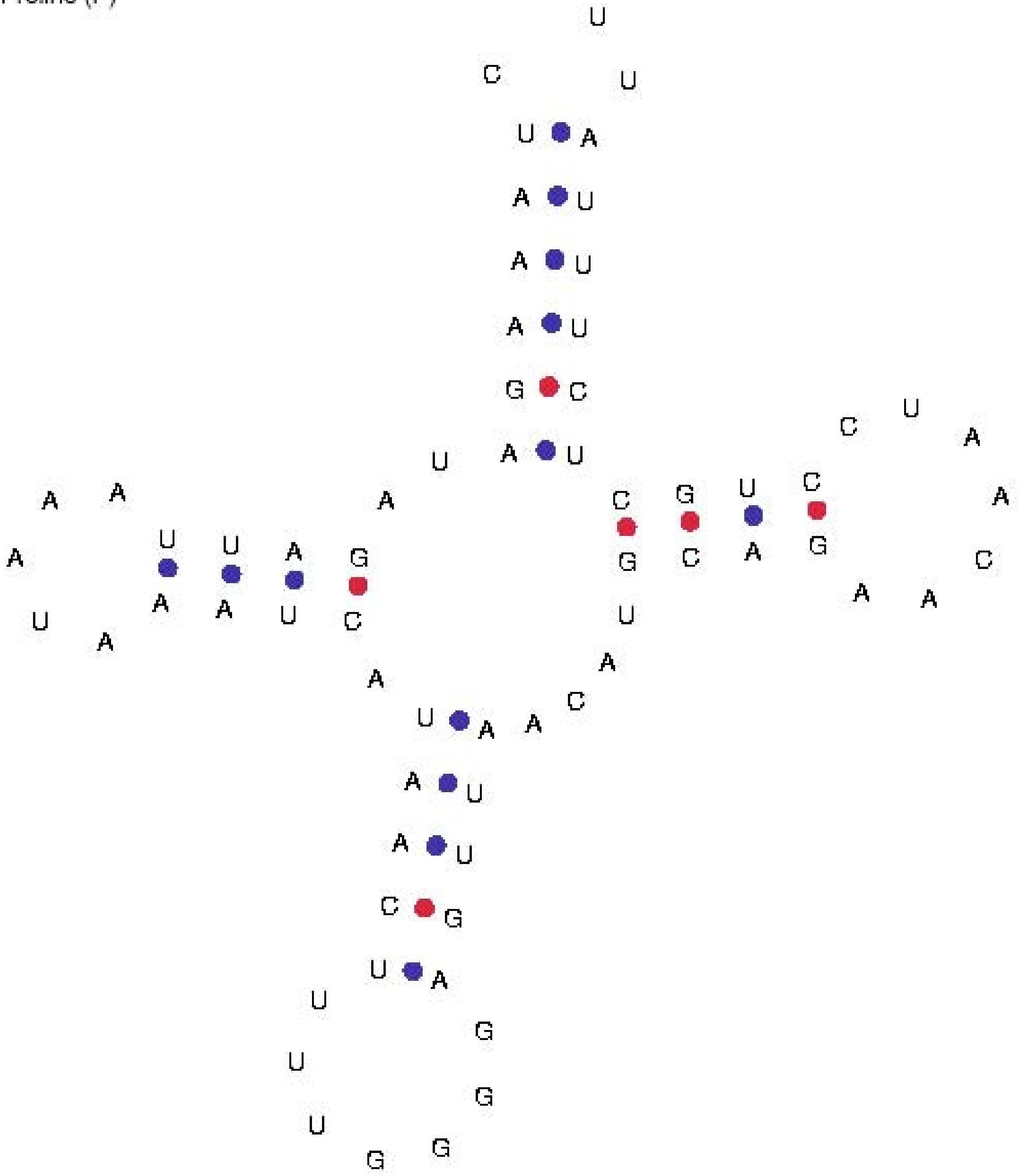

Serine (S1)
(ucu)

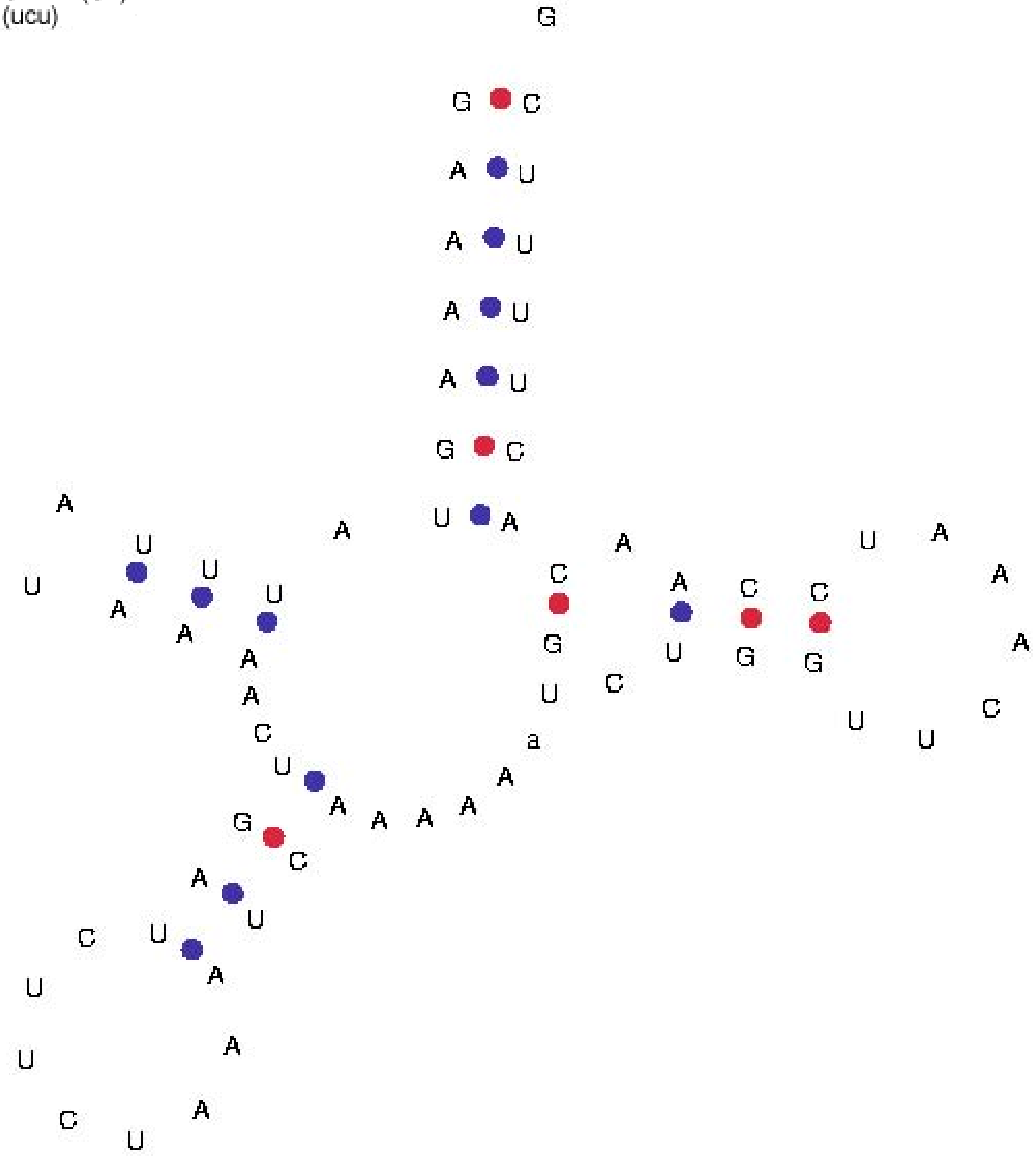

Serine (S2)
(uga)

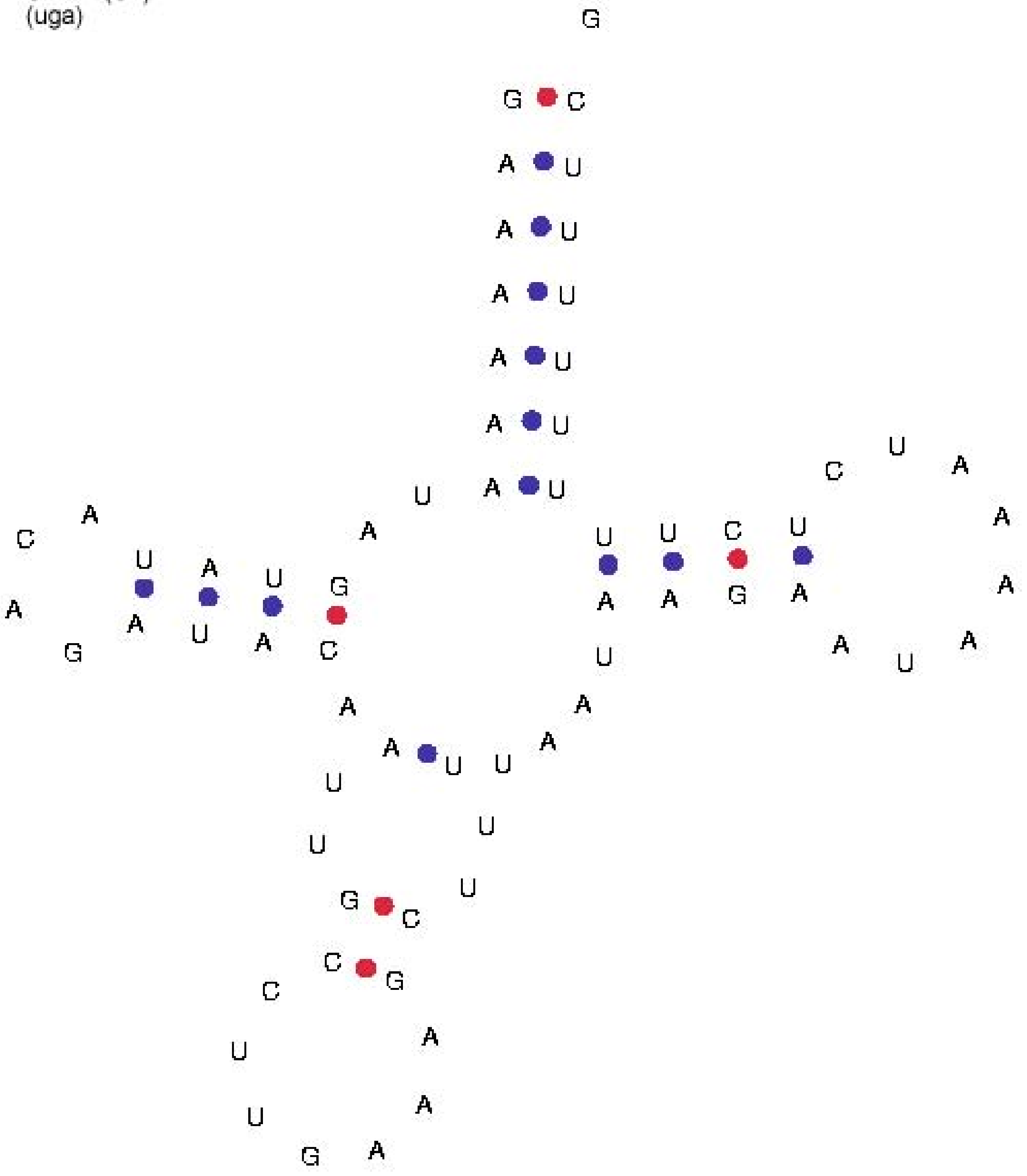

Tryptophan (W)

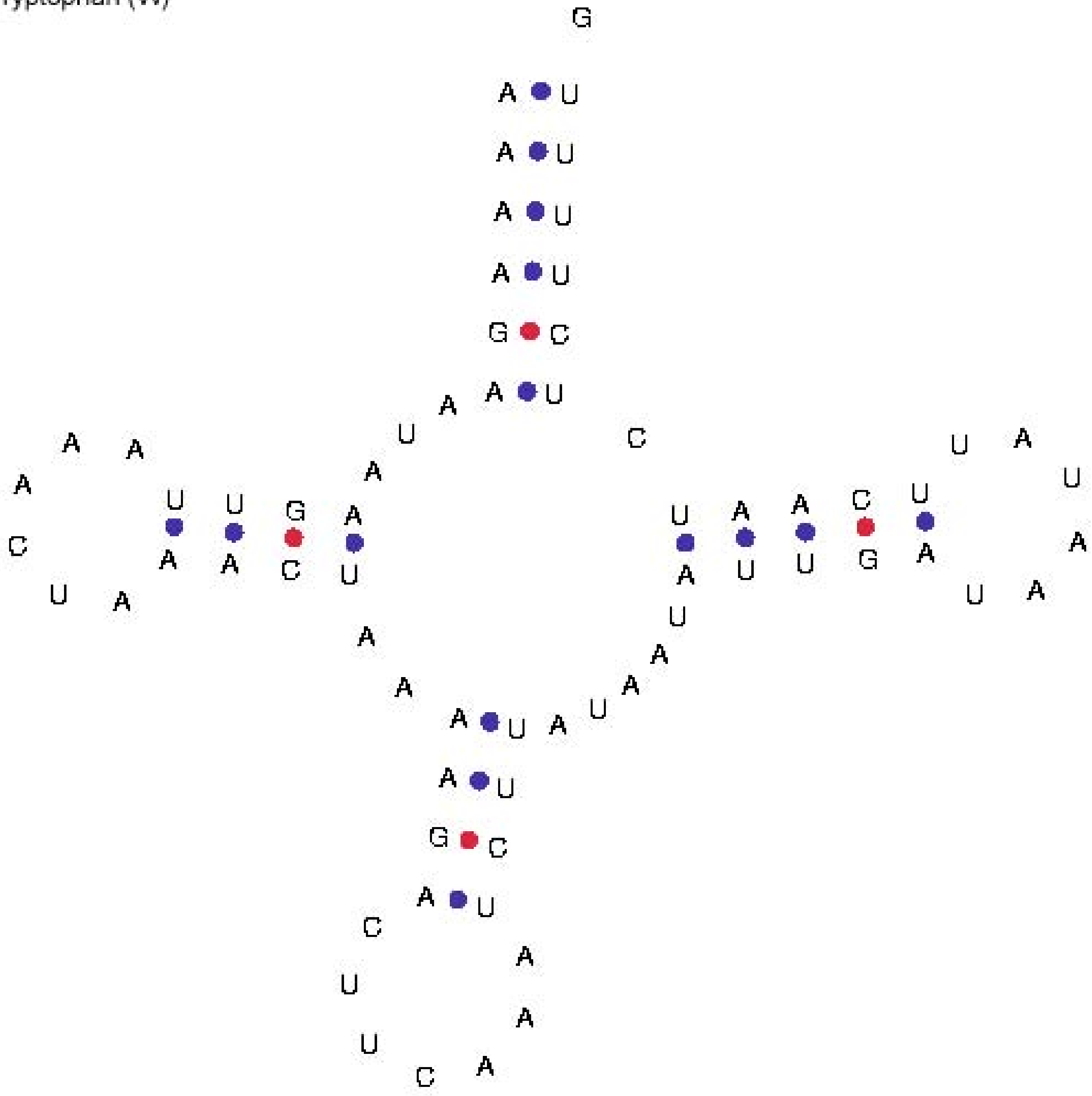

Tyrosine (Y)

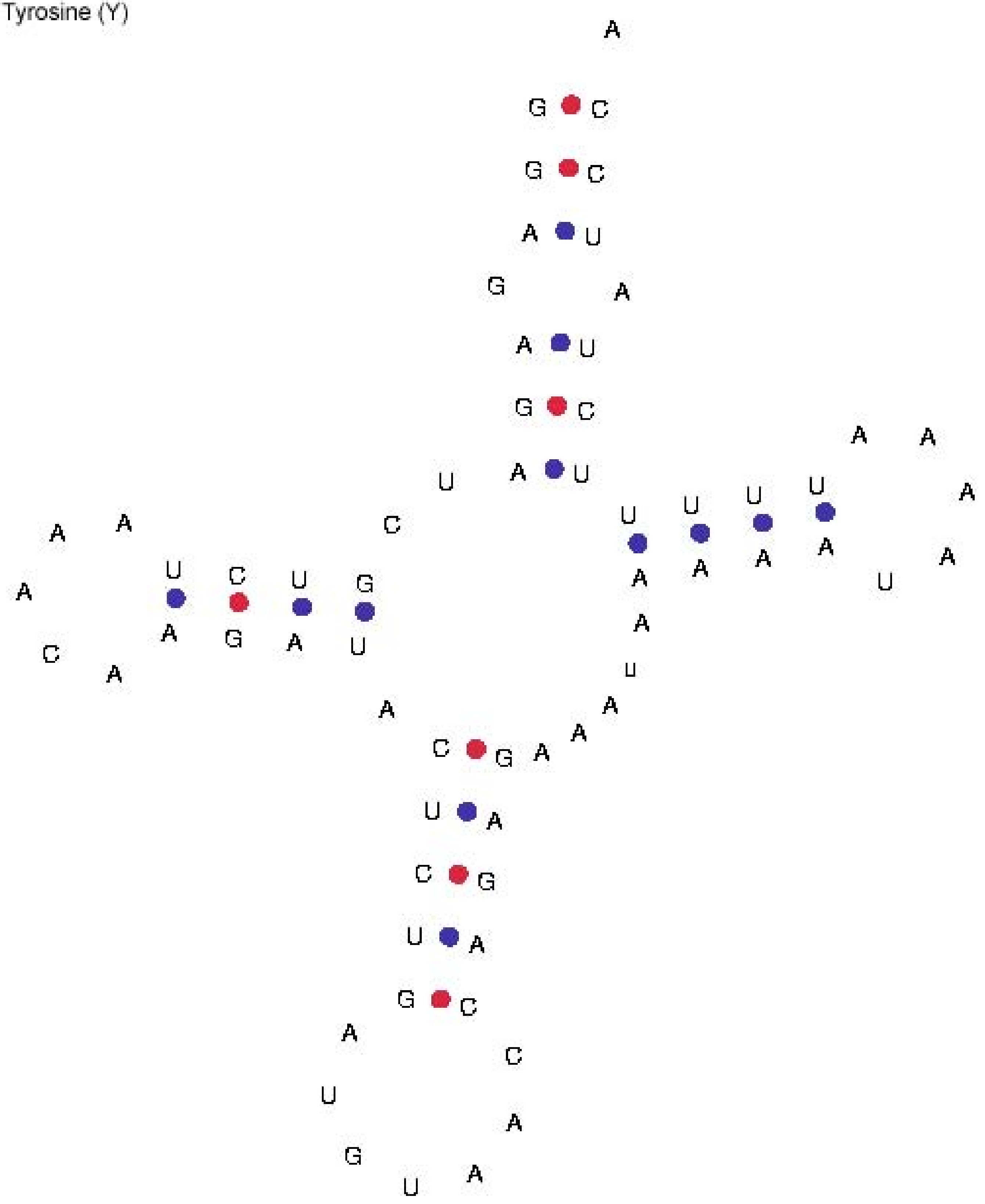

Valine (V)

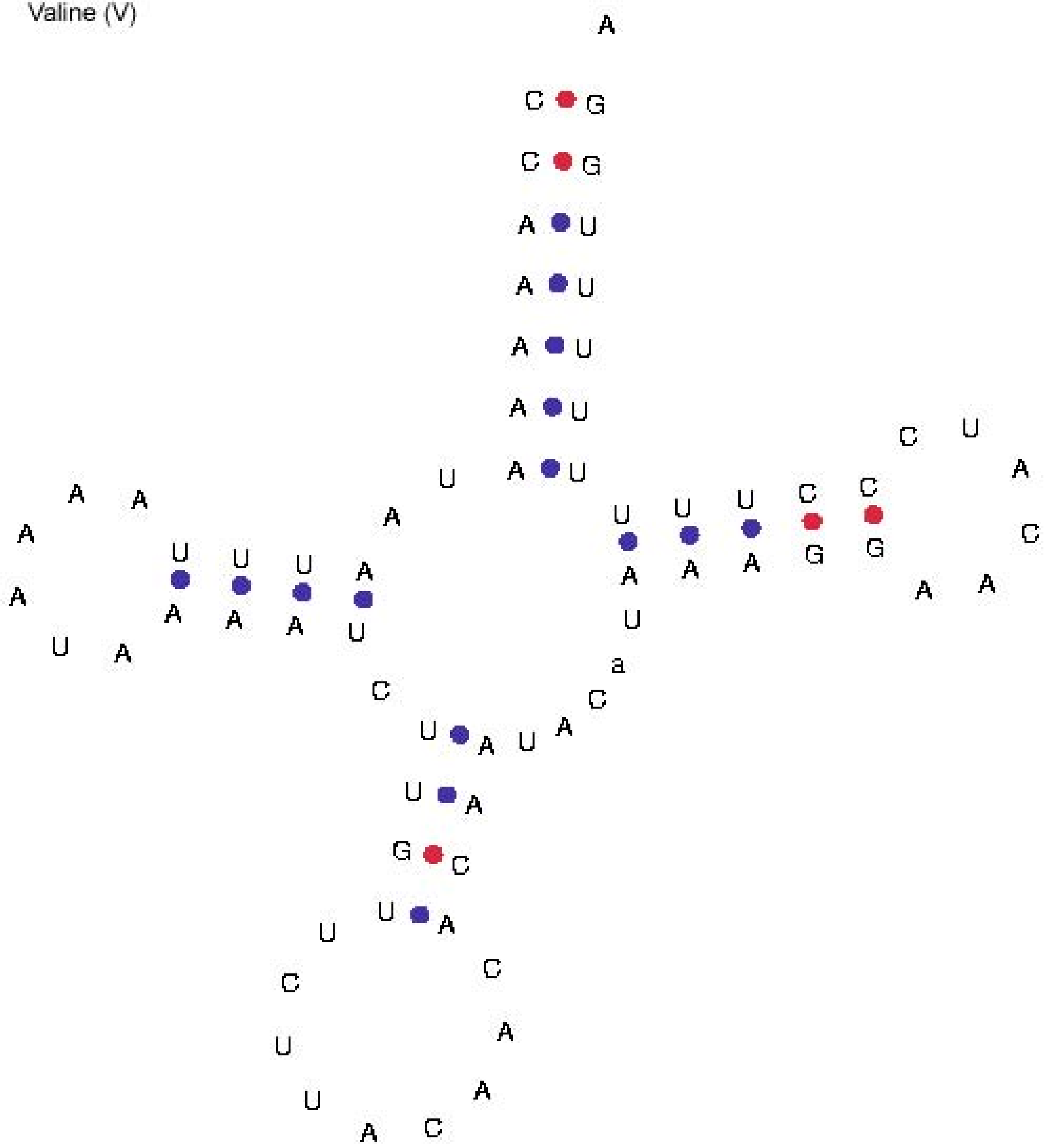

Supplement: Additional file 1 — A. vastus mtDNA tRNA structures. Structure diagrams of all 18 A. vastus mtDNA encoded tRNAs predicted by tRNAscan-SE. [file 1471-2164-9-33-S1.pdf]
